# Precipitated Adrenal Insufficiency by Osilodrostat and Fluconazole in Ectopic Adrenocorticotrophic Hormone Syndrome With a Pituitary Microadenoma and Cryptococcus Infection

**DOI:** 10.1016/j.aed.2026.02.001

**Published:** 2026-02-05

**Authors:** Kristen Lee, Carolina Mendes Pessoa, Wenyu Huang

**Affiliations:** Division of Endocrinology, Metabolism and Molecular Medicine, Northwestern University Feinberg School of Medicine, Chicago, Illinois

**Keywords:** ectopic ACTH syndrome, pituitary tumor, Cryptococcus infection

## Abstract

**Background/Objective:**

Ectopic adrenocorticotropic hormone (ACTH) syndrome (EAS) is a challenging condition, particularly when the primary tumor cannot be localized. This case report described a patient with EAS and a concomitant pituitary tumor who developed adrenal crisis while receiving osilodrostat for EAS and fluconazole for cryptococcal infection before bilateral adrenalectomy.

**Case Report:**

A 27-year-old man presented with progressive fatigue, proximal muscle weakness, resistant hypertension, and weight gain. Laboratory tests showed significant hypercortisolemia. Magnetic resonance imaging showed a 5 mm pituitary tumor, but inferior petrosal sinus sampling confirmed ectopic ACTH source. Given the severity of hypercortisolism, osilodrostat was initiated. Subsequently, a positron emission tomography scan revealed a hypermetabolic right lung nodule. Biopsy confirmed the nodule to be a cryptococcus infection for which fluconazole was started. Due to failure to locate the primary tumor, bilateral adrenalectomy was planned. Two weeks after starting fluconazole, the patient developed adrenal insufficiency prompting hospital admission. Osilodrostat was held and stress dose steroid was initiated. He underwent bilateral adrenalectomy and had significant improvement in his clinical status.

**Discussion:**

This case presented a patient with EAS and a concomitant pituitary tumor. Osilodrostat was used before bilateral adrenalectomy. After discovery of a rare opportunistic fungal infection, antifungal treatment was initiated which appeared to have synergistic action with osilodrostat in suppressing cortisol production.

**Conclusion:**

Presence of a pituitary tumor in ACTH-dependent hypercortisolemia does not always signify Cushing disease. Concomitant use of adrenal steroidogenesis inhibitors may precipitate adrenal crisis.

**Highlights**

• The diagnosis of Cushing syndrome due to ectopic adrenocorticotropic hormone (ACTH) syndrome (EAS) is often challenging, particularly when there is a pituitary tumor but the primary source of ectopic ACTH cannot be localized
• The combination of a pituitary microadenoma and ACTH-dependent hypercortisolism does not always conform to Cushing disease. Inferior petrosal sinus sampling is helpful in making the correct diagnosis
• Bilateral adrenalectomy can be considered for EAS absent of clear source of ACTH
• For severe EAS, medical treatment for hypercortisolism can be used to optimize the clinical status of the patient before planned surgery
• Combined use of adrenal steroidogenesis inhibitors may have synergistic effect, resulting in accelerated reduction in cortisol production and adrenal insufficiency

**Clinical Relevance**

This case highlights the challenges of diagnosing ectopic adrenocorticotropic hormone (ACTH) syndrome, particularly when the primary source of ACTH cannot be localized. The immunocompromised nature in ectopic ACTH syndrome is exemplified by the rare cryptococcal infection. There is potential synergistic effect of fluconazole and osilodrostat on the inhibition of adrenal steroidogenesis, precipitating adrenal insufficiency.

## Introduction

Ectopic adrenocorticotropic hormone (ACTH) syndrome (EAS) is a rare cause of hypercortisolism, accounting for 10% to 15% of cases of endogenous Cushing syndrome.^1^ Clinical course of EAS can be rapidly progressive and its sequalae may lead to life-threatening complications including opportunistic infections, thromboembolism, and cardiovascular events.^2^^,^^3^

The diagnostic approach to EAS can be complex. It should begin with biochemical testing for endogenous hypercortisolism through a combination of 24-hour urine free cortisol, late night salivary cortisol, and 1-mg overnight dexamethasone suppression test.^4^ Upon confirmation of hypercortisolism, a morning serum ACTH level can differentiate between ACTH dependence and independence with a high or inappropriately normal ACTH level indicating ACTH dependence.^5^ Once ACTH dependence is established, the source of ACTH production must be localized. Cushing disease as result of a pituitary corticotroph tumor is the most common cause of endogenous hypercortisolism making up 60% to 70% of all cases.^6^ Pituitary magnetic resonance imaging (MRI) can help localization of ACTH source. However, a notable limitation is that a corticotroph adenoma may not be shown on pituitary imaging and a pituitary incidentaloma can be observed on MRI in a patient with EAS.^4^^,^^7^ Inferior petrosal sinus sampling (IPSS) remains the “gold standard” for differentiating Cushing disease from EAS.^5^^,^^8^ When IPSS is suggestive of EAS, localizing the source can sometimes be difficult. EAS is most commonly localized in the lung; however, if the size is small <1.5 cm or in close proximity to lung vasculature it can be challenging to identify.^4^ Despite the limitations of identifying EAS localization, the severity of EAS can necessitate treatment without source localization.

To reduce the morbidity and mortality related to EAS, reducing cortisol level is paramount either via medical management or surgery. Blocking cortisol synthesis is a cornerstone of therapy. Osilodrostat rapidly decreases cortisol production by inhibiting the 11β-hydroxylase and has been used to reduce cortisol level in EAS.^9^ Additionally, imidazole derivative such as ketoconazole, commonly used as antifungal treatment, can inhibit several key enzymes in adrenal steroidogenesis and have also been successfully used to treat endogenous Cushing syndrome.^10^^,^^11^

Here we describe a patient with EAS who while being treated with osilodrostat before bilateral adrenalectomy developed adrenal crisis during concurrent treatment with fluconazole for opportunistic cryptococcal infection.

## Case Report

A 27-year-old man presented to endocrinology clinic with progressive fatigue, proximal muscle weakness, worsening depression, anxiety, insomnia, headaches, blurred vision, and a 30 kg weight gain in the last 2 years. He also described a 1-year history of type 2 diabetes mellitus which was poorly controlled with metformin, dulaglutide, and insulin glargine and a A1C of 9.6% (81 mmol/mol), and uncontrolled hypertension requiring amiloride, carvedilol, spironolactone, and valsartan. Eight months prior to presentation to our clinic, he was hospitalized for severe hypokalemia with K 2.8 mmol/L (reference range 3.3-5.1 mmol/L). Work-up was negative for primary aldosteronism but notable for an elevated 24-hour urine free cortisol 3,376.3 μg/24 h (reference range 4-50 μg/24 h). At the time he was discharged on spironolactone and referred to endocrinology clinic. He followed up 4 months later and had repeat 24-hour urine free cortisol which was also elevated at 554.6 μg/24 h, prompting referral to endocrinology clinic. His family history was negative for pituitary tumor or Cushing syndrome.

On physical examination, blood pressure was 140/90 mm Hg, pulse rate 130/min, weight 104.3 kg, height 188 cm, and body mass index 28.89 kg/m^2^. Central obesity, abdominal purple striae, ecchymoses, facial plethora, proximal muscle weakness, buffalo hump, and supraclavicular fat pad were noted. Laboratory findings summarized in Table 1 showed white blood cell count of 11.32 K/μL (reference range 4.00-10.00 K/μL), glucose 459 mg/dL (reference range 70-100 mg/dL), potassium 3.4 mmol/L (reference range 3.3-5.1 mmol/L) while on spironolactone, A1C 7.7% (61 mmol/mol), baseline ACTH 142 pg/mL (reference range 7.2-63.6 pg/mL), morning cortisol 58.1 μg/dL (reference range 5.0-25.0 μg/dL), and dehydroepiandrosterone sulfate) 956 μg/dL (reference range 167-591 μg/dL). Serum cortisol post 8-mg overnight dexamethasone suppression test was 6.5 μg/dL. Other pituitary hormone labs showed thyroid-stimulating hormone 0.48 μIU/mL (reference range 0.40-4.00 μIU/mL), free T4 0.67 ng/dL (reference range 0.7-1.50 ng/dL), prolactin 18.3 ng/mL (reference range 2.6-13.1 ng/mL), and insulin-like growth factor 161 (reference range 63-373 ng/mL).

**Table 1:** Biochemical Evaluation at Presentation to Endocrine Clinic

Laboratory test	Result	Normal range
am cortisol	58.1 μg/dL	5.0-25.0 μg/dL (137.94-689.70 nmol/L)
ACTH	122 pg/mL	7.2-63.6 pg/mL
DHEA-sulfate	956 μg/dL	167-591 ug/dL
24-h urine free cortisol	3,376 μg/24 h	4.0-50.0 μg/24 h (11.036-137.95 nmol/24 h)
Cortisol after 8-mg dexamethasone suppression test	6.5 μg/dL	N/A
Total testosterone	177 ng/dL	250-1,100 ng/dL
Free testosterone	43.9 pg/mL	35.0-155.0 pg/mL
TSH	0.73 μIU/mL	0.40-4.00 μIU/mL
FT4	0.67 ng/dL	0.70-1.50 ng/dL
Prolactin	18.3 ng/mL	2.6-13.1 ng/mL
Insulin-like growth factor 1	161 ng/mL	63-373 ng/mL
LH	2.0 mIU/L	1.2-9.0 mIU/L
FSH	6.2 mIU/L	1.3-19.3 mIU/L
Plasma renin activity	13.11 ng/mL/h	0.25-5.82 ng/mL/h
Aldosterone (LC-MS/MS)	<1 ng/dL	≤28 ng/dL
A1C	7.7%	4.0%-5.6%
Glucose	459 mg/dL	65-100 mg/dL(3.61-5.55 mmol/L)
White blood count	11.32 K/μL	4.00-10.00 K/μL

Abbreviations: ACTH = adrenocorticotrophic hormone; DHEA = dehydroepiandrosterone; FSH = follicle-stimulating hormone; FT4 = free thyroxine; LC-MS/MS = liquid chromotography-tandem mass spectrometry; LH = luteinizing hormone; TSH = thyroid-stimulating hormone.

MRI pituitary showed a 5 mm pituitary adenoma. However, IPSS showed no central to peripheral gradient of ACTH both pre-desmopressin (<2.0) and post-desmopressin (< 3.0) administration, and no apparent ACTH increase in response to desmopressin, all consistent with an ectopic source of ACTH (Table 2). Extensive imaging was done to locate the primary lesion for EAS. A computed tomography (CT) scan of chest/abdomen/pelvis was negative for malignancy. Fluorodeoxyglucose-positron emission tomography (PET) scan showed a hypermetabolic nodule in the right lung. Subsequently, DOTA-0-Tyr3-octreotide PET/CT failed to reveal a neuroendocrine tumor. Biopsy of the right lung nodule confirmed cryptococcus infection.

**Table 2:** IPSS Results

ACTH (pg/mL)			
t (min)	R IPS	L IPS	IVC
–10	198	195	178
–5	200	194	180
1	199	198	172
3	213	205	190
5	208	193	198
10	194	203	188
15	201	192	195
30	193	185	188

Prolactin (ng/mL)			
t (min)	R IPS	L IPS	IVC
–10	316.7	41.4	37.3
–5	258.7	38.2	34.2
*Mean*	287.7	39.8	35.75

Abbreviations: ACTH = adrenocorticotrophic hormone; IPS = inferior petrosal sinus; IPSS = inferior petrosal sinus sampling; IVC = inferior vena cava.

Given the severity of hypercortisolism and occult primary lesion, bilateral adrenalectomy was recommended, and he was initiated on osilodrostat 3 mg twice a day before surgery. Deep vein thrombosis prophylaxis was also started. Two weeks after starting osilodrostat, the patient reported symptoms of fatigue, dizziness, and decreased appetite. Laboratory tests were notable for elevated morning cortisol 34.6 μg/dL (reference range 5.0-25.0 μg/dL), ACTH 192 pg/mL (reference range 7.2-63.6 pg/mL), and 24 urine free cortisol 798 (reference range 5-64). Given concern for patient reported steroid withdrawal symptoms, osilodrostat dose was initially reduced. However, after further discussion with the patient it became clear his symptoms were more likely attributable to uncontrolled Cushing syndrome; thus, the dose was increased to 4 mg twice a day. His 24-hour urine free cortisol improved but remained elevated at 508 μg/24 h (reference range 5-64 μg/24 h), leading to further dose increase to 5 mg twice a day. Three weeks after this dose increase, his morning cortisol improved to 20.1 μg/dL (reference range 5.0-25.0 μg/dL) and 24-hour urine free cortisol was 316.3 μg/24 h.

Two and a half months after initiation of osilodrostat, due to new discovery of cryptococcus infection, fluconazole was initiated. Two weeks after start of fluconazole, he presented with abdominal pain, nausea/vomiting, diarrhea, and proximal weakness. Vital signs showed blood pressure of 104/72 mm Hg, and pulse rate of 126/min. Laboratory findings showed Na 131 mmol/L (reference range 133-146 mmol/L), K 5.4 mmol/L (reference range 3.5-5.1 mmol/L), glucose 128 mg/dL (reference range 65-100 mg/dL), alanine aminotransferase 190 U/L (reference range 0-5 U/L), aspartate aminotransferase 88 (reference range 0-39 U/L), and triglyceride 2,827 mg/dL (normal, < 100 mg/dL). CT scan of chest/abdomen/pelvis showed no evidence of new acute infection or hemorrhage. Fluconazole was initially held given hepatocellular injury; however, liver enzymes improved prior to discontinuation of this medication. Random cortisol on admission was 0.5 μg/dL (reference range 5.0-25.0 μg/dL). Given evidence of adrenal insufficiency due to concurrent use of osilodrostat and fluconazole, osilodrostat was held on admission and the patient was initiated on stress dose hydrocortisone (HC) 50 mg IV every 6 hours. The patient’s symptoms and tachycardia improved, and HC was gradually tapered. He then underwent bilateral adrenalectomy. Pathology showed left adrenal gland with hyperplastic change and right gland with hyperplastic change and focal hemorrhage. After bilateral adrenalectomy, he was initiated on HC and fludrocortisone. For hypertriglyceridemia, he was started fenofibrate and atorvastatin. He was discharged on HC dose 50 mg in the morning, 30 mg in the afternoon, and 10 mg in the evening due to concern for ongoing glucocorticoid withdrawal symptoms. Fluconazole was restarted hospital day 2 through discharge. See Figure for a summary of the diagnosis and treatment course of EAS.

**Fig:**
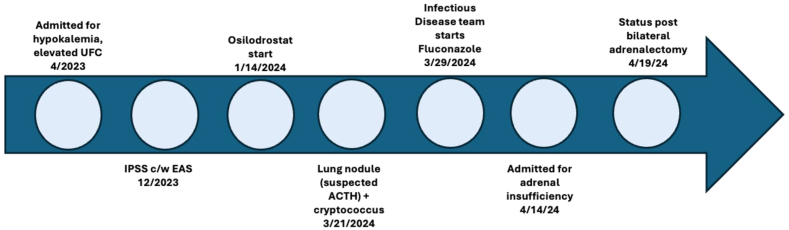
Timeline of significant clinical events from diagnosis through bilateral adrenalectomy. *ACTH* = adrenocorticotropic hormone; *EAS* = ectopic adrenocorticotropic hormone syndrome; I*PSS* = inferior petrosal sinus sampling; *UFC* = 24-hour urine free cortisol.

### Outcome and Follow-Up

On outpatient follow-up, HC was gradually tapered to physiologic replacement dose over 13 months. The patient was instructed to continue fluconazole upon hospital discharge to complete a 3- to 6-month course but he declined to do so. Repeat cryptococcus antigen test was negative. The patient was able to discontinue all diabetes medications and all but one antihypertensive medication. Repeat DOTA-0-Tyr3-octreotide PET/CT while the patient was receiving physiologic replacement of glucocorticoid was again negative for neuroendocrine tumor.

## Discussion

Here we present a case of a 27-year-old man with ACTH-dependent Cushing syndrome and a 5-mm pituitary tumor. However, IPSS ultimately revealed his excessive ACTH was ectopic in nature. This case underscores the importance of IPSS in differentiating between Cushing disease and EAS despite positive pituitary MRI, especially in the case of a small pituitary lesion.^8^ Although Cushing disease is the most common etiology of ACTH-dependent Cushing syndrome, his clinical presentations raised suspicion for EAS due to the rapid onset and severity of hypercortisolism with very high ACTH levels.^12^

EAS represents up to 15% of endogenous Cushing syndrome cases. Identifying the ectopic source can be challenging despite extensive imaging. The most common site is the lungs due to pulmonary neuroendocrine tumors and small cell lung carcinoma.^8^^,^^13^ In approximately 20% of these cases, the ectopic ACTH producing lesion remains occult such as in this case despite repeat imaging.^14^

Interestingly in our case, the PET scan employed to localize the source of ectopic ACTH tumor led to a lung nodule biopsy that was notable for opportunistic cryptococcal infection. Cushing syndrome is associated with immune suppression that predispose patients to opportunistic infections.^15^ EAS and its association with immunocompromised state have been described in a dose dependent manner such that the greater severity of hypercortisolism the higher risk of infection which at times can be fatal.^15^ In a cohort of 90 patients with EAS, 51% of patients had infections.^6^ The most commonly reported disseminated fungal infections in patients with EAS include *Pneumocystis, Cryptococcus, Nocardia,* and *Aspergillosis.*^16^

Our patient was treated with fluconazole for cryptococcus infection. In a cautionary tale, this inadvertent combined use of adrenal steroidogenesis inhibitors osilodrostat for Cushing syndrome management and fluconazole for fungal infection led to rapid decline of cortisol and development of adrenal insufficiency. Osilodrostat inhibits 11β-hydroxylase, the key enzyme responsible for the final step in cortisol synthesis and indicated to treat endogenous Cushing syndrome.^17^^,^^18^ Fluconazole is an azole antifungal that is not approved for the treatment of hypercortisolism. However, it shares similar properties with ketoconazole. Both ketoconazole and fluconazole inhibit multiple enzymes in cortisol synthesis including 11β-hydroxylase and 17-α-hydroxylase. Of note there have been several case reports documenting adrenal suppression in the setting of fluconazole.^19^ Unique to this case, the combination of osilodrostat and fluconazole appeared to have additive/synergistic effect on adrenal steroidogenesis inhibition resulting in adrenal crisis.

This case demonstrates the importance of IPSS in the differential diagnosis of ACTH-dependent Cushing syndrome in the presence of pituitary tumor. This case also highlights the complexity of EAS including various complications from it including cryptococcus infection. Our case also underscores the combined effect on adrenal steroidogenesis when fluconazole and osilodrostat are used concurrently.

## Conclusion

This patient has both a pituitary tumor and ACTH-dependent Cushing syndrome but through IPSS was found to have EAS. It is important to remember that a pituitary tumor and evidence of ACTH-dependent hypercortisolism does not automatically mean Cushing disease. For patients with EAS, osilodrostat is an effective modality to quickly reduce cortisol, improve the metabolic sequalae of hypercortisolism, and optimize a patient’s clinical status before surgery. Caution should be given to precipitating adrenal insufficiency with the synergic action of osilodrostat and treatment of opportunistic fungal infection with a triazole like fluconazole or imidazole derivative. Glucocorticoid replacement should be used for steroid withdrawal symptoms or adrenal insufficiency. The immunocompromised nature of Cushing syndrome can lead to life-threatening complication and the timing of initiating antifungal prophylaxis has yet to be elucidated.

## Patient Consent

We thank our patient for allowing us to share his case and the entire care team for their dedication to caring for this patient. The authors have obtained written consent.

## Disclosure

Consulting fee; Self; W.H., honoraria from consulting and speaking received from Recordati Rare Diseases.

## References

[1] Hernández I., Espinosa-de-los-Monteros A.L., Mendoza V. (2006). Ectopic ACTH-secreting syndrome: a single center experience report with a high prevalence of occult tumor. Arch Med Res.

[2] Blew K., Van Mater D., Page L. (2023). Successful management of Cushing syndrome from ectopic ACTH secretion in an adolescent with osilodrostat. JCEM Case Rep.

[3] Ejaz S., Vassilopoulou-Sellin R., Busaidy N.L. (2011). Cushing syndrome secondary to ectopic adrenocorticotropic hormone secretion: the University of Texas MD Anderson Cancer Center Experience. Cancer.

[4] Ceccato F., Barbot M., Mondin A., Boscaro M., Fleseriu M., Scaroni C. (2023). Dynamic testing for differential diagnosis of ACTH-dependent Cushing syndrome: a systematic review and meta-analysis. J Clin Endocrinol Metab.

[5] Alba E.L., Japp E.A., Fernandez-Ranvier G. (2022). The Mount Sinai clinical pathway for the diagnosis and management of hypercortisolism due to ectopic ACTH syndrome. J Endocr Soc.

[6] Ilias I., Torpy D.J., Pacak K., Mullen N., Wesley R.A., Nieman L.K. (2005). Cushing’s syndrome due to ectopic corticotropin secretion: twenty years’ experience at the National Institutes of Health. J Clin Endocrinol Metab.

[7] Testa R.M., Albiger N., Occhi G. (2007). The usefulness of combined biochemical tests in the diagnosis of Cushing’s disease with negative pituitary magnetic resonance imaging. Eur J Endocrinol.

[8] Elenius H., Nieman L.K. (2025). Recognition and management of ectopic ACTH secreting tumors. J Endocr Soc.

[9] Bessiène L., Bonnet F., Tenenbaum F. (2021). Rapid control of severe ectopic Cushing’s syndrome by oral osilodrostat monotherapy. Eur J Endocrinol.

[10] Tabarin A., Navarranne A., Guérin J., Corcuff J.B., Parneix M., Roger P. (1991). Use of ketoconazole in the treatment of Cushing’s disease and ectopic ACTH syndrome. Clin Endocrinol (Oxf).

[11] Herrera-Martínez A.D., Feelders R.A., de Herder W.W. (2019). Effects of ketoconazole on ACTH-producing and non-ACTH-producing neuroendocrine tumor cells. Horm Cancer.

[12] Gadelha M., Gatto F., Wildemberg L.E., Fleseriu M. (2023). Cushing’s syndrome. Lancet.

[13] Alexandraki K.I., Grossman A.B. (2010). The ectopic ACTH syndrome. Rev Endocr Metab Disord.

[14] Hayes A.R., Grossman A.B. (2018). The ectopic adrenocorticotropic hormone syndrome: rarely easy, always challenging. Endocrinol Metab Clin North Am.

[15] Hasenmajer V., Sbardella E., Sciarra F., Minnetti M., Isidori A.M., Venneri M.A. (2020). The immune system in Cushing’s syndrome. Trends Endocrinol Metab.

[16] Graham B.S., Tucker W.S. (1984). Opportunistic infections in endogenous Cushing’s syndrome. Ann Intern Med.

[17] Fleseriu M., Biller B.M.K. (2022). Treatment of Cushing’s syndrome with osilodrostat: practical applications of recent studies with case examples. Pituitary.

[18] (2025). Recordarti. Isturisa. https://www.accessdata.fda.gov/drugsatfda_docs/label/2025/212801s003lbl.pdf.

[19] Burns K., Christie-David D., Gunton J.E. (2016). Fluconazole in the treatment of Cushing’s disease. Endocrinol Diabetes Metab Case Rep.

